# La manucure et la pédicure dans la ville de Ouagadougou (Burkina Faso): pratiques et risqué

**DOI:** 10.11604/pamj.2016.24.109.8641

**Published:** 2016-05-31

**Authors:** Nina Korsaga-Somé, Jean Baptiste Andonaba, Muriel Sidnoma Ouédraogo, Gilbert Patrice Tapsoba, Léopold Ilboudo, Cérina Savadogo, Fatou Barro-Traoré, Pascal Niamba, Adama Traoré

**Affiliations:** 1 Service de Dermatologie-Vénéréologie CHU Yalgado Ouédraogo Ouagadougou, Burkina Faso; 2 Service de Dermatologie, Vénéréologie CHU SouroSanou Bobo-Dioulasso, Burkina Faso; 3 Unité de Formation et Université de Ouagadougou, Burkina Faso; 4 Université Polytechnique de Bobo-Dioulasso, Burkina Faso

**Keywords:** Manucure-pédicure, pratiques, risques, Ouagadougou, Manucure-pedicure, practices, risks, Ouagadougou

## Abstract

La manucure-pédicure est l'ensemble des soins esthétiques des mains, des pieds et des ongles. Au Burkina Faso, l'usage des produits de manucure-pédicure, les techniques utilisées ainsi que les risques encourus restent méconnus. L'objectif de notre étude était d’évaluer la pratique de la manucure-pédicure dans la ville de Ouagadougou. Nous avons mené une étude transversale descriptive de décembre 2010 à novembre 2012 incluant tout les praticiens ayant au moins six mois d'expérience dans l'activité et les clients présents sur les lieux au moment de l'enquête. Nous avons interrogé au total 313 praticiens et 313 clients. L’âge moyen des praticiens était de 19 ans et celui des clients de 32,2 ans. Les praticiens fixes étaient en majorité des femmes (96,87%), ceux mobiles surtout des hommes (68,37%), et 64,53% des clients étaient des femmes. Le pourcentage de praticiens n'ayant pas reçu de formation professionnelle était de 93,92%. 29,7% des praticiens faisaient tremper les instruments pendant au moins dix minutes dans de l'eau de javel; 75,71% savaient que l'utilisation de certains outils étaient dangereux et 26,51% étaient avaient présenté des effets secondaires. Parmi les clients, 40,25% savaient que le matériel utilisé comportait des risques et 30,35% avaient été victimes d'accidents. Les soins de manucure et de pédicure se font dans les salons de coiffure par des coiffeuses non formées à l'exercice de la profession La provenance et la composition des produits n'est pas connues. Des produits non recommandés sont utilisés (shampooing pour trempage des pieds, lame de rasoir et ciseaux pour raclage des pieds). Le recours à la manucure et/ou pédicure est parfois nécessaire mais cela ne doit pas faire perdre de vue les risques encourus. Une sensibilisation des clients et une formation des praticiens semblent nécessaires pour minimiser les risques.

## Abstract (en)

Pedicure-manicure represents the aesthetic care of hands, feet and nails. In Burkina Faso, the use of manicure-pedicure products, the techniques used and the level of risk remain unknown. The aim of our study was to evaluate the practice of manicure-pedicure in the city of Ouagadougou. We conducted a descriptive cross-sectional study of all practitioners with at least six months experience in aesthetic care and customers present at the time of the survey from December 2010 to November 2012. We interviewed a total of 313 practitioners and 313 clients. The average age of practitioners was 19 years and of customers was 32.2 years. Fixed location practitioners were mostly women (96.87%) while mobile practitioners were mostly men (68.37%); 64.53% of customers were women. The percentage of practitioners who did not receive professional training was 93.92%. 29.7% of practitioners soaked the instruments in javel water for at least ten minutes; 75.71% knew that the use of certain tools was dangerous and 26.51% had side effects. 40.25% of customers knew that the used equipment may pose some risks and 30.35% were victims of accidents. The manicure and pedicure is done in hair salons by untrained hairdressers to the professional practice. The origin and composition of the products is not known. Not recommended products are used (foot soak shampoo, razor blade and scissors for feet scraping). The use of manicure and/or pedicure is sometimes necessary but that should not obscure the risks to which it exposes customers. Customers education and practitioners training seem necessary to minimize risks.

## Introduction

Selon le Petit Larousse illustré 2012 [1], le manucure est la “personne chargée des soins esthétiques des mains, des ongles”, tandis que la manucure est “l′ensemble des soins esthétiques des mains, des ongles”. Le pédicure est “le professionnel paramédical qui effectue les soins des pieds”. Nous pouvons alors définir la manucure/pédicure effectuée dans les salons de beauté de la ville de Ouagadougou comme l'ensemble des soins esthétiques des mains, des pieds et des ongles. Il s'agit de soins se faisant dans un milieu humide avec le plus souvent partage d'objets tranchants. Lors des soins de l′ongle surtout, lorsque l′éponychium (la cuticule) est retiré, les instruments utilisés en manucure/pédicure entrent en contact avec le sang des clients [2]. Il existe donc un risque de transmission de micro-organismes. En effets plusieurs études font état du partage de matériel de manucure et de pédicure comme facteur de risque associé à l'infection par le virus de l'immunodéficience humaine (VIH) et le virus de l'hépatite C (VHC) [2–5]. Bien que beaucoup de clients fréquentent les établissements de beauté [2, 4], peu de données sont disponibles sur les pratiques et les risques dans ces établissements. Cette pénurie de données n′est pas nécessairement liée à un manque d′événements, mais est plutôt liée à l′absence relative de notification et d’études épidémiologiques menées pour ce type d′activité. Au Burkina Faso, aucune étude ne s'est encore penchée sur les conditions d'exercice de cette profession. Le but de notre étude était de combler cette lacune en identifiant les acteurs intervenant dans cette profession, les sites ou cadre d'exercice, ainsi que les pratiques et les risques auxquels les intervenants et les bénéficiaires s'exposent.

## Méthodes

**Type d′étude, lieu et période:** Notre étude était de type transversal à visée descriptive. Elle s'est déroulée de décembre 2010 à novembre 2012.

**Echantillonnage:** Compte tenu des ressources limitées, nous avons procédé à un choix raisonné selon le type et le niveau de fréquentation des salons. Nous avons distingué deux procédés d'exercice du métier: la manucure/pédicure fixe exercée dans les salons de coiffure et la manucure/pédicure pratiquée en ambulatoire. La ville de Ouagadougou était subdivisée en 30 secteurs. En tenant compte de la superficie des secteurs, nous avons d'abord identifié les 5 plus grands secteurs. Dans ces 5 plus grands secteurs, nous avons choisi de façon raisonnée 5 salons de coiffure par secteur, soit un total de 25 salons de coiffure. Dans les 25 autres secteurs restants qui étaient de plus petite superficie, nous avons choisi 3 salons de coiffure par secteur, soit un total de 75 salons de coiffure. Le nombre global de salons de coiffure retenus était donc de 100. Pour les ambulants nous avons exploré la ville de Ouagadougou en choisissant d'interroger tous les praticiens ambulants que nous allions rencontrer avec leur client du moment, durant la période de collecte.

**Matériel:** Nous avons choisi d'interroger 1 praticien et 1 client par salon fixe, ce qui faisait un total de 100 praticiens et 100 clients de salons fixes. Nous avons pu interroger 213 praticiens et 213 clients ambulants. Pour ne pas interroger doublement les clients et biaiser les résultats de l'analyse, nous avons noté leur adresse (nom, prénom, numéro de téléphone, secteur).

**Variables étudiées:** La collecte des données était effectuée par observation suivie d′une entrevue de face à face auprès des praticiens et des clients. Les données étaient colligées sur un questionnaire qui comportait les variables suivantes: les caractéristiques sociodémographiques des praticiens et des clients: âge, sexe, niveau de scolarisation; le cadre d'exercice: salon fixe, salon mobile, propreté, espace pour la circulation des clients, système d’élimination des déchets, aération, présence de source d'eau; la pratique: formation et connaissance sur les risques, les produits utilisés, les techniques; les risques: la prévention des infections (ports de gants, élimination déchets tranchants, stérilisation des instruments), les incidents et accidents.

**Critères d′inclusion:** Ils étaient pour les praticiens, avoir une durée d'activité d'au moins six mois, et pour les clients, être présent le jour de l'enquête sur un site de pédicure/manucure et accepter de répondre au questionnaire, après un consentement éclairé verbal. L'analyse des données était effectuée avec les logiciels Epi Info 3.5.1 2008 et Excel 2007.

**Définitions opérationnelles:** Nous avons considéré comme praticiens fixes tous ceux ou celles qui pratiquaient la manucure/pédicure dans un cadre précis et reconnu comme lieu de pratique (salons et institut de beauté). Les praticiens mobiles étaient ceux qui pratiquaient en ambulatoire dans la rue. Nous avons considéré comme clients fixes tous les clients fréquentant un salon de beauté et comme clients mobiles, ceux qui recevaient des services de manucure/pédicure dans la rue. Nous avons considéré comme salon propre, tout salon dans lesquels le plancher, la toiture, le plafond et les murs n′étaient pas tachés, ni souillés et qui étaient régulièrement nettoyés. L'exiguïté du salon était basée sur deux aspects: l'espace pour la circulation des clients et le nombre de places assises. L'aération était appréciée sur le nombre de portes et de fenêtres ainsi que la présence ou non d'un ventilateur. Nous avons considéré comme produits et matériels non recommandés pour la manucure/pédicure, les solutions d'origine inconnues à base de chlorure de sodium, soude caustique, eau, neutralisant; les shampooing pour cheveux; les savons liquides fabriqués localement à base de concentré d'hydroxyde de soude, de sel, de colorant et de parfum et utilisé pour le nettoyage des maisons et des meubles; le matériel tranchant comme les filets en fer, les boites percées, les couteaux, les ciseaux et les lames.

## Résultats

Nous avons interrogé au total 313 praticiens et 313 clients dont 100 praticiens fixes et 213 praticiens en ambulatoire, et 100 clients fixes et 213 clients en ambulatoire.

**Les caractéristiques sociodémographiques des praticiens et des clients:** Lâge moyen des praticiens était de 19 ans, la tranche d’âge de [25-35]était la plus représentée avec 40% des effectifs Tableau 1. L’âge moyen des clients était de 32,2 avec pour âges extrêmes 15 et 60 ans Tableau 1. Les praticiens fixes étaient en majorité des femmes (96,87%), ceux mobiles surtout des hommes (68,37%), et 64,53% des clients étaient des femmes. Les praticiens mobiles étaient de nationalité non burkinabé dans 78,42% des cas. La répartition des praticiens et des clients selon le niveau de scolarisation est représentée par le Tableau 2.

**Tableau 1:** Répartition des 313 praticiens et 313 clients selon la tranche d’âge et la fréquence

Tranches d’âge/an	PRATICIENS		CLIENTS	
	Fixes N^+^ et%	Mobiles N^+^ et%	Fixes N^+^ et%	Mobiles N^+^ et%
[15-25]	36 (36)	77 (36)	30 (30)	85 (40)
[25-35]	40 (40)	85 (40)	32 (32)	62 (29)
[35-45]	16 (16)	51 (24)	26 (26)	34 (16)
[45-55]	08 (08)	00 (00)	12 (12)	32 (15)
**Total**	**100 (100)**	**213 (100)**	**100 (100)**	**213 (100)**

N^+^ = effectif

**Tableau 2:** Répartition des 313 praticiens et 313 clients selon la le niveau d'instruction et la fréquence

Niveau d'instruction	PRATICIENS		CLIENTS	
	Fixes N^+^ et%	Mobiles N^+^ et%	Fixes N^+^ et%	Mobiles N^+^ et%
Non alphabétisé	09 (09)	87 (40,84)	09 (09)	83 (38,96)
A^+ +^ en langue nationale	07 (07)	19 (08,92)	08 (08)	39 (18,32)
Primaire	34 (34)	53 (24,88)	11 (11)	66 (30,98)
Secondaire	47 (47)	54 (25,35)	37 (37)	25 (11,74)
Universitaire	03 (03)	00 (00,00)	35 (35)	00 (00,00)
**Total**	**100 (100)**	**213 (100)**	**100 (100)**	**213 (100)**

N^+^ = effectif, A^+^ = Alphabétisé

**Le cadre d′exercice:** Concernant les salons fixes, 57% étaient assez propres, 58% avaient un espace restreint, 97,3% comportaient au moins une porte et une fenêtre. Un ventilateur était présent dans 43% de ces salons. Parmi les 100 praticiens interrogés, tous jetaient les objets tranchants dans la poubelle, 46 jetaient les eaux usées sur la voie publique, 15 dans les caniveaux et 8 dans les canaux d’évacuation. Les pratiques en ambulatoire se faisaient dans la rue, au milieu des gens dans un espace bien réduit.

**La pratique de la pédicure/manucure:** Le pourcentage de praticiens qui n'avaient pas reçus de formation professionnelle était de 93,92%. Parmi les praticiens fixes 75,71% savaient que l'utilisation de certains outils était dangereuse et 18,3% avait une assez bonne maitrise des différentes techniques. Cent pour cent des praticiens mobiles ne savaient pas que le matériel qu'ils utilisaient comportait des risques. Le pourcentage de clients qui savaient que le matériel utilisé comportait des risques était de 40,25%. Les produits non recommandés pour la manucure étaient utilisés par 74,87% des praticiens et pour la pédicure par 45,84% d'entre eux, comme les ciseaux pour le raclage des pieds par exemple. Seulement 2,23% utilisait des produits vendus en pharmacie pour la pédicure. La colle forte liquide était utilisée comme fixateur pour la pose d′ongles artificiels. Les Tableau 3 et Tableau 4 présentent techniques, les produits et les matériels utilisés pour la manucure et la pédicure.

**Tableau 3:** Répartition des différentes techniques et matériels utilisés pour la manucure selon la fréquence

Techniques, produits et matériels	Praticiens mobiles N^+^ = 213	Praticiens Fixes N^+^ = 100
Port de gants	00	00
**Trempage des mains:**		
Pâte pour manucure	00	64
Savon liquide	00	57
Shampooing pour cheveux	00	18
**Frottement:**		
Pierre ponce	00	88
Brosse pour main	00	62
Filet en caoutchouc dur	00	03
**Raclage:**		
Ciseaux	00	72
Lame de rasoir	05	64
Repoussoir	00	79
Stick	00	09
Pince à envies	00	13
**Limage:**		
Lime en carton	00	67
Lime métallique	00	07
Lame de rasoir	00	19
**Vernissage:**		
Base	00	05
Vernis	196	98

N^+^ = effectif

**Tableau 4:** Répartition des différentes techniques etmatériels utilisés pour la pédicure selon la fréquence

Techniques, produits et matériels	Praticiens mobiles N^+^ = 213	Praticiens Fixes N^+^ = 100
Port de gants	00	00
**Trempage des pieds:**		
Antiseptique moussant	00	13
Pâte pour pédicure	00	78
Shampooing pour cheveux	00	67
**Frottement:**		
Pierre ponce	00	98
Brosse pour pied	00	94
**Filet en fer**	00	**03**
Boite percée	00	**17**
Râpe	00	**53**
**Raclage:**		
**Ciseaux**	**39**	**41**
**Lame de rasoir**	**15**	**67**
Repoussoir	00	79
Stick	00	09
**Pince à envies**	00	**13**
**Limage:**		
Lime métallique	00	05
**Vernissage:**		
Base	00	05
Vernis	00	98
**Fixateur (Colle)**	00	**14**

N^+^ = effectifs

**Figure 1:**
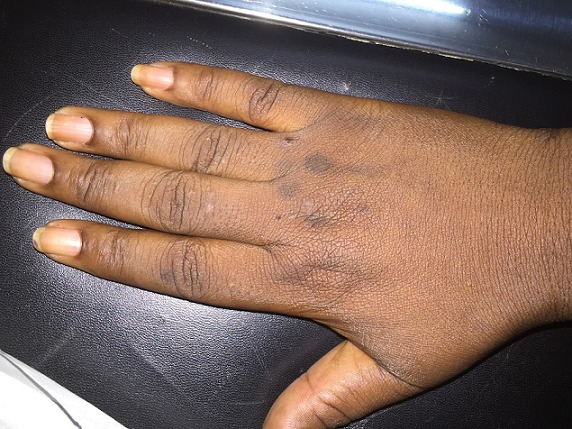
Xanthonychie distale (couleur jaun-âtre de la partie distale de la tablette unguéale) après pose d'ongles artificiels. (Source: collection service de Dermatologie-Vénéréologie du CHU Yalgado Ouédraogo de Ouagadougou)

**Figure 2:**
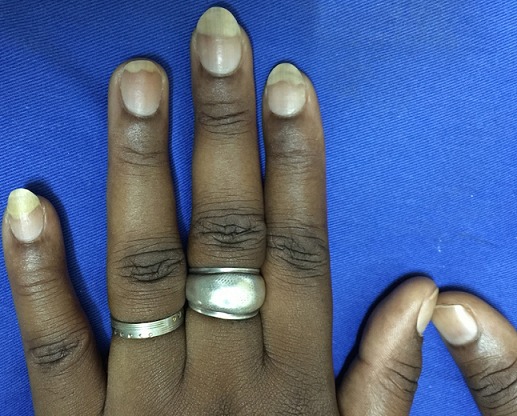
Leuconychie distale (blanchiment de la partie distale de la tablette unguéale) après pose d'ongles artificiels. (Source: collection service de Dermatologie-Vénéréologie du CHU Yalgado Ouédraogo de Ouagadougou)

**Figure 3:**
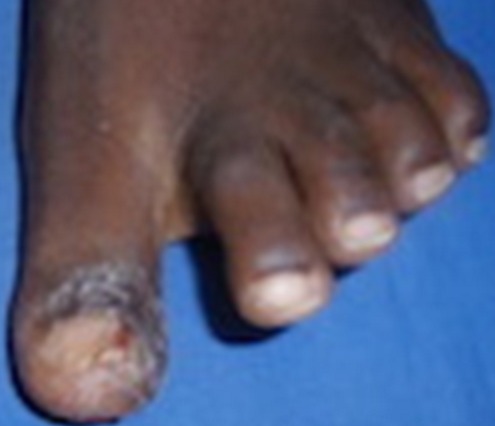
Eczéma périunguéale (squamo-croûteux) du pouce gauche après une séance pédicure. (Source: collection service de Dermatologie-Vénéréologie du CHU Yalgado Ouédraogo de Ouagadougou)

**Les risques:** Concernant la prévention des infections, aucun praticien ne portait de gants (Tableau 3,Tableau 4). Aucun praticien n'utilisait d'instruments jetables. Aucun praticien ne se lavait non plus les mains avant et après la séance de manucure/pédicure. Sur aucun des sites d'exercice il n'y avait de boite à tranchants, l’élimination déchets tranchants se faisait dans la poubelle. Seulement 29,7% des praticiens fixes faisaient tremper les instruments pendant au moins dix minutes dans de l'eau de javel. Aucun praticien mobile ne stérilisait le matériel avant usage. Parmi les praticiens 26,51% signalaient un accident, dont une piqure/coupure par un objet tranchant (22%), une irritation (36,76%), une dermite caustique (35%) et un eczéma (25%). Parmi les clients, 30,35% étaient victimes d'accidents dont 7,99% de piqure/coupure par un objet tranchant, 6,70% de dermite caustique et 7,35% d'eczéma de contact. La pose d'ongles artificiels chez les clients avait engendré un changement de couleur (69%) (Figure 1, Figure 2), un traumatisme (36%), un décollement (33%), un eczéma périunguéal (15%) (Figure 3), une ulcération préiunguéale (14%), un effritement (07%) et une chute de l'ongle (10%).

## Discussion

**Limites de l’étude:** La pratique de la manucure/pédicure dans les salons de beauté est favorable à la transmission de micro-organismes du fait du partage d'objets tranchants [3]. Les limites financières ne nous permettaient pas de faire le dépistage des infections à VIH et à VHC comme d'autres auteurs l'on fait pour évaluer ce risque [2–5].

**Les caractéristiques générales:** Notons que dans la ville de Ouagadougou, les soins de manucure/pédicure se font dans des salons de coiffure par des coiffeurs non spécialisés pour ce travail. Ailleurs les cadres d'exercice étaient de vrais salons de manicure/pédicure [2–5]. Dans notre contexte, les clients et les praticiens sont donc exposés au risque d′inhalation de produits renfermant de l'acide thioglycolique ou ses sels, destinés à friser, défriser et onduler les cheveux [6]. Il en est de même des produits contenant du toluène ou du phtalate de di-butyle (BDP) [7]. Le manque d'exigence juridique sur une scolarité minimum pourrait expliquer le bas niveau de scolarisation des praticiens. Ce constat était également fait par Garbaccio et coll [3]. Il s'agit par ailleurs d'une profession où l'apprentissage se fait plus sur le tas plutôt que dans des écoles spécialisées. Nous pensons que les autorités devraient exiger un minimum certification pour l'exercice de cette profession.

**La pratique de la pédicure/manucure:** Le manque de formation [3] pourrait être à l'origine d'une sous-estimation des risques et de pratiques inadéquates. La méconnaissance des risques était plus élevée chez les praticiens en ambulatoire que chez ceux des salons de beauté. Les techniques de trempage, suivie de raclage avec des objets tranchants (lame, filet de fer, ciseaux (Figure 3), boite percée, couteau) constituaient un gros risque de transmission de micro-organismes. De plus, les produits utilisés étaient soit inadaptés (shampooing pour cheveux), soit de composition ou de provenance inconnu. La colle utilisée pour la pose d'ongle artificiel était constituée principalement de cyanoacrylate de méthyl qui est un adhésif tenace et qui pourrait se révéler dangereux pour l'homme [8]. Notons quele parabène contenu dans les shampooing, et autres produits cosmétiques expose également à des risques très importants [9], de même le formaldéhyde, le toluène ou le phtalate de di-butyle (DBP) présents dans des vernis à ongles utilisés [10]. Le formaldéhydeprésent dans les shampoings, les produits pour durcir les ongles était classé en juin 2004 comme « cancérigène certain » [11]. Un approvisionnement en pharmacie aura l'avantage de prévenir ces risques.

**Les risques:** Notre enquête montrait que seuls les praticiens des salons fixes étaient conscients des risques liés à leur profession et prenaient quelques précautions très insuffisantes dans la prévention des infections (absence totale de port de gants, de boite à tranchants, de lavage des mains, très peu de désinfection). Les praticiens ambulants réutilisaient le même matériel non désinfecté sur plusieurs clients. Ils estimaient que les ciseaux, les coupe-ongles et les repoussoirs ne touchaient pas le corps du client et ne pouvaient donc pas être source de contamination. Le niveau de gravité de l'acte posé ou la négligence de la part du praticien ou du client pose de sérieux problèmes face à la pandémie du SIDA. Plusieurs autres études rapportaient que le respect des normes de biosécurité était insuffisant lors des travaux de manucure/pédicure [2, 4]. Les autorités devraient se pencher sur la question en exigeant un minimum de formation et de recyclage. Les effets secondaires de la manucure/pédicure à type de piqûre/coupure, dermite caustique et allergique que nous avons notés étaient également rapportés par plusieurs études, qui signalaient également la manucure pédicure comme facteur de risque de transmission d'infection bactérienne, fongique et virale comme l'infection à VIH et à VHC [2–5, 12–14]. La manucure et la pédicure étaient également notées comme facteurs de risque de transmission du VHC chez les donneurs de sang dans la ville de Belem en Amazonie brésilienne [15]. Les risques de transmission de micro-organisme existent bien lors de ces bien travaux. Aussi les praticiens que les clients devraient être sensibilisés sur ces risques.

## Conclusion

Cette étude nous a permis de donner quelques éléments de réponses aux questions que nous nous étions posé. Ainsi nous avons pu noté que les acteurs de la manucure/pédicure exerçant dans les salons ou instituts de beauté de Ouagadougou étaient surtout de jeunes femmes ayant un niveau de scolarisation du primaire ou du secondaire; et que ceux mobiles étaient surtout des hommes non burkinabé. Ces acteurs n'avaient pas non plus reçu de formation spécialisée. Le cadre d'exercice, les techniques et les produits utilisés n’étaient pas souvent appropriés. La prévention des infections était insuffisante et plusieurs accidents étaient rapportés. Les risques existaient aussi bien pour les praticiens que les clients et étaient liés aussi bien aux produits qu'aux techniques utilisées. Des campagnes d'information et de sensibilisation, de formation et de recyclage permettraient d'améliorer cette pratique dans le but de minimiser ces risques dans la ville de Ouagadougou.

### Etat des connaissances actuelles sur le sujet

- Les soins de manucure et de pédicure comportent des risques pour les clients et les praticiens;
- Des mesures de prévention doivent être prises comme le port de gants, le lavage des mains et l’évitement d'objets tranchants.

### Contribution de notre étude à la connaissance

- Les soins de manucure et de pédicure se font dans les salons de coiffure par des coiffeuses non formées à l'exercice de la profession et qui ne respectent pas les mesures de prévention;
- La provenance et la composition des produits ne sont pas connues;
- Des produits non recommandés sont utilisés (shampooing pour trempage des pieds, usage d'objets tranchants comme lame de rasoir et ciseaux pour raclage des pieds).
